# Evaluating methods for identifying and quantifying *Streptococcus pneumoniae* co-colonization using next-generation sequencing data

**DOI:** 10.1128/spectrum.03643-23

**Published:** 2024-11-05

**Authors:** Jada Hackman, Martin L. Hibberd, Todd D. Swarthout, Jason Hinds, James Ashall, Carmen Sheppard, Gerry Tonkin-Hill, Kate Gould, Comfort Brown, Jacquline Msefula, Andrew A. Mataya, Michiko Toizumi, Lay-Myint Yoshida, Neil French, Robert S. Heyderman, Stefan Flasche, Brenda Kwambana, Stéphane Hué

**Affiliations:** 1 Faculty of Epidemiology and Population Health, Department of Infectious Disease Epidemiology, The London School of Hygiene and Tropical Medicine, London, United Kingdom; 2 Department of Pediatric Infectious Diseases, Institute of Tropical Medicine, Nagasaki University, Nagasaki, Japan; 3 School of Tropical Medicine and Global Health, Nagasaki University, Nagasaki, Japan; 4 Faculty of Infectious and Tropical Diseases, The London School of Hygiene and Tropical Medicine, London, United Kingdom; 5 Research Department of Infection, Division of Infection and Immunity, University College London, London, United Kingdom; 6 Department of Epidemiology, Julius Center for Health Sciences and Primary Care, University Medical Centre Utrecht, Utrecht, the Netherlands; 7 BUGS Bioscience, London Bioscience Innovation Centre, London, United Kingdom; 8 Institute for Infection and Immunity, St George’s University of London, London, United Kingdom; 9 Vaccine Preventable Bacteria Section, UK Health Security Agency (UKHSA), London, United Kingdom; 10 Department of Biostatistics, University of Oslo, Blindern, Norway; 11 Malawi Liverpool Wellcome Research Programme, Blantyre, Malawi; 12 Institute of Infection, Veterinary and Ecological Sciences, University of Liverpool, Liverpool, United Kingdom; 13 Department of Clinical Sciences, Liverpool School of Tropical Medicine, Liverpool, United Kingdom; Johns Hopkins Medicine, Baltimore, Maryland, USA

**Keywords:** co-carriage, pneumococcus, Africa, *Streptococcus pneumoniae*, sequencing, microarray, serotyping

## Abstract

Detection of multiple pneumococcal serotype carriage can enhance monitoring of pneumococcal vaccine impact, particularly among high-burden childhood populations. We assessed methods for identifying co-carriage of pneumococcal serotypes from whole-genome sequences. Twenty-four nasopharyngeal samples were collected during community carriage surveillance from healthy children in Blantyre, Malawi, which were then serotyped by microarray. Pneumococcal DNA from culture plate sweeps were sequenced using Illumina MiSeq, and genomic serotyping was carried out using SeroCall and PneumoKITy. Their sensitivity was calculated in reference to the microarray data. Local maxima in the single-nucleotide polymorphism (SNP) density distributions were assessed for their correspondence to the relative abundance of serotypes. Across the 24 individuals, the microarray detected 77 non-unique serotypes, of which 42 occurred at high relative abundance (>10%) (per individual, median, 3; range, 1–6 serotypes). The average sequencing depth was 57X (range: 21X–88X). The sensitivity of SeroCall for identifying high-abundance serotypes was 98% (95% CI, 0.87–1.00), 20% (0.08–0.36) for low abundance (<10%), and 62% (0.50–0.72) overall. PneumoKITy’s sensitivity was 86% (0.72–0.95), 20% (0.06–0.32), and 56% (0.42–0.65), respectively. Local maxima in the SNP frequency distribution were highly correlated with the relative abundance of high-abundance serotypes. Six samples were resequenced, and the pooled runs had an average fourfold increase in sequencing depth. This allowed genomic serotyping of two of the previously undetectable seven low-abundance serotypes. Genomic serotyping is highly sensitive for the detection of high-abundance serotypes in samples with co-carriage. Serotype-associated reads may be identified through SNP frequency, and increased read depth can increase sensitivity for low-abundance serotype detection.

**IMPORTANCE:**

Pneumococcal carriage is a prerequisite for invasive pneumococcal disease, which is a leading cause of childhood pneumonia. Multiple carriage of unique pneumococcal serotypes at a single time point is prevalent among high-burden childhood populations. This study assessed the sensitivity of different genomic serotyping methods for identifying pneumococcal serotypes during co-carriage. These methods were evaluated against the current gold standard for co-carriage detection. The results showed that genomic serotyping methods have high sensitivity for detecting high-abundance serotypes in samples with co-carriage, and increasing sequencing depth can increase sensitivity for low-abundance serotypes. These results are important for monitoring vaccine impact, which aims to reduce the prevalence of specific pneumococcal serotypes. By accurately detecting and identifying multiple pneumococcal serotypes in carrier populations, we can better evaluate the effectiveness of vaccination programs.

## INTRODUCTION

Carriage of *Streptococcus pneumoniae* (Sp) is a prerequisite for invasive pneumococcal disease (IPD), and IPD is a leading cause of childhood pneumonia. Most pneumococci are encapsulated with a complex capsular polysaccharide (cps) that contributes to its virulence and pathogenicity (1). All typeable pneumococci are typed by the *cps* locus flanked by the *dexB* and *aliA* genes (2, 3), and there are more than 100 distinct serotypes identified (4). Non-typeable pneumococci, often non-invasive, are serologically non-typeable due to the lack of functional capsular operon (5). Currently available vaccines against Sp include the 23-valent pneumococcal polysaccharide-based vaccine (PPV23) and three polysaccharide–protein conjugate vaccines (PCV10, PCV13, PCV15, PCV20) where PCV13 is recommended for children under 5 years old and PCV15 or PCV20 for adults over 65 years old (6).

Carriage of multiple unique pneumococcal serotypes at a single time point (co-carriage) is common among children in settings with high carriage prevalence (7), with an estimated 40% of children found to carry multiple serotypes within their first year of life in Gambia and Malawi (8–10) and 47% of infants in Papua New Guinea (11). Moreover, carriage of multiple serotypes provides an opportunity to exchange *cps* locus via recombination and thus can lead to serotype switching and result in vaccine escape (12, 13). Monitoring of co-carriage is an important part of surveillance activities, including in the characterization of the response of pneumococci to vaccine pressures. Due to the complexities of co-carriage, studies have mostly relied on reporting single serotype carriage from a representative genome from purified single-colony picks, which often identifies the serotype in greater relative abundance, e.g., the dominant serotype. Additionally, among co-carriage, relatively low-abundance vaccine-type serotypes (8%) have been reported to go undetected (10), thus, limiting the accuracy of carriage surveillance and underestimating individual serotype carriage rates and vaccine impact (14).

Additionally, there needs to be a greater understanding of co-carriage and the contribution of low-abundance serotypes to transmission, likely due to a limited sensitivity in detecting minor variants from genomic data. Carriage of multiple sequence serotypes further increases the within-host bacteria diversity. With the potential for unsampled lineages from either the source or recipient, this further complicates work to infer the transmission direction in the presence of a strong transmission bottleneck where a single strain is transmitted from the source to the recipient (15). To resolve this limitation, adequate sampling at the collection and sequencing steps would be needed for both the source and recipient of the transmission.

The Quellung reaction is considered the gold standard for serotyping pneumococcus using a non-genomic-based approach. Quellung uses serotype-specific antibodies where the pneumococcal isolates are sequentially tested first with a pooled antisera and then against each antisera (16). Because this method is labor intensive, it requires training and expertise and is not practically scalable to large studies (16), the Quellung reaction is primarily used by reference laboratories (17). Latex sweep, latex agglutination from a sweep of colonies, is a commonly used serotyping method due to its being cost effective and field deployable at detecting pneumococcal co-carriage; however, this method is limited to detecting serotypes at >25% relative abundance (14).

DNA microarray is another technique with high sensitivity and specificity for molecular serotyping and detection of pneumococcal co-carriage based on the detection of *cps* genes in DNA extracts from samples (18–20). Microarray has been previously validated as the gold standard for pneumococcal serotype detection based on spiked samples and has shown superiority against all other methods with 99% sensitivity and 100% positive predictive value for the detection of minor serotypes in the spiked samples (18). Microarray is rapid, differentiates all known serotypes, and can be used to determine the relative abundance of serotypes in instances of co-carriage of multiple serotypes (21). However, this method requires trained personnel and specialized equipment, and logistical and cost requirements can be an obstacle to implement at sites with limited capacity.

Whole-genome sequencing (WGS) has become an increasingly cost-effective alternative for serotyping single isolates from carriage samples (i.e., from blood, cerebrospinal fluid, or other normally sterile sites) in routine disease (22). WGS can offer additional insights, such as genetic relatedness of diseases or antibiotic resistance, in addition to serotyping. Bioinformatic tools, such as PneumoCaT and SeroBA, use a k-mer-based method to identify concordance between query cps locus next-generation sequencing reads and the pneumococcus Capsular Type Variant database (CTVdb) (22), with high sensitivity (99% and 98%, respectively). However, these tools were developed for IPD surveillance (assumed to be caused by a single serotype) and thus have limited capacity to identify multiple serotypes in co-carriage. However, the recently developed pipelines PneumoKITy (23) and SeroCall (24) identify serotypes in co-carriage with high sensitivity (>85%), making them an attractive alternative to previous approaches in carriage surveillance. Both methods rely on the PneumoCaT CTVdb; however, SeroCall uses a mapping approach, while PnuemoKITy uses a k-mer-based approach which offers higher speed (23, 24).

In this study, genomic serotyping results, including SeroCall, PneumoKITy, and a novel approach using single-nucleotide polymorphism (SNP) frequency distributions were compared to microarray for identifying serotypes in co-carriage and the capacity to differentiate these serotypes for further analyses.

## MATERIALS AND METHODS

### Sample collection and processing

Samples were collected as part of a larger prospective observational study, the study design and sample collection of which were previously detailed (25). Briefly, nasopharyngeal swabs were collected from asymptomatic children during a study in Blantyre, Malawi, to evaluate the impact of the 2011 introduction of the 13-valent pneumococcal conjugate vaccine (PCV13) on carriage and disease. Upon sample collection, nasopharyngeal swabs were immediately stored in skim milk–tryptone–glucose–glycerin (STGG) medium and stored at −80°C within 8 hours. Aliquots of STGG were cultured on a selective medium (COBA, Oxoid, UK) and genomic DNA extracted from plate sweeps for microarray analysis as previously described (10) then stored at −20°C.

### Sample selection

Children were recruited from the community using random household sampling, and a subset of 57 samples was selected to represent a range of multiple serotype carriage; from those, 24 samples were anonymized and sequenced for this study. The 24 Sp-positive nasopharyngeal swab samples that were included in this study were selected to represent a mix of colonization with a range of one to six pneumococcal serotypes present at varying relative abundances, as previously determined by microarray (Table S1). The serotyping results from the microarray array are the reference serotyping method for this study.

### Sequencing and sequence processing

The residual DNA extracts from the 24 samples used for the prior microarray analyses were also processed for WGS at the London School of Hygiene and Tropical Medicine (London, United Kingdom) to prevent potential variation due to culture and sample preparation. Whole-genome sequencing was done on the Illumina MiSeq platform using a Qiagen FX library kit (Qiagen, location), with enzymatic fragmentation for 12 min targeting 300- to 400-bp fragments. Six of the 24 samples were selected to be resequenced at a higher sequencing depth. The selection was based on increasing read depth for future work on haplotype reconstruction (sample ID S03), improving sequencing read depth from the original sequencing run (S22) and increasing read depth to increase the sensitivity of genomic serotyping methods to detect low-abundance serotypes (S09, S11, S16, S19).

Adaptors from the raw data were trimmed using Trimmomatic v0.39 (26). The forward and reverse FASTQ files containing the reads were aligned using the reference genome KK0981, with Burrow–Wheeler Alignment v.0.7.17 (BWA-MEM) and SAMtools mpileup v1.9.114 (27). The quality of the sequencing data was assessed using Kraken2, and non-Sp reads were excluded from the analysis of the SNP densities and frequencies but not from the genomic serotyping methods. Sequencing coverage and depth were calculated from mapped reads in the bam files containing only Sp reads, using Samtools. For the six samples resequenced at greater depth, original and resequencing reads were pooled, resulting in higher sequencing depth.

### Genomic serotyping

Two genomic serotyping tools were used, SeroCall (24) and PneumoKITy (23), to identify the occurrence of co-carriage. These were carried out using the sequencing raw reads (e.g., not filtered for Sp reads). There were no options to modify the SeroCall algorithm; however, PneumoKITy was initially run with the default parameters, including the requirement that 90% of k-mers were found in the reference. This was later lowered to 80% and 70% to investigate the corresponding trade-offs in sensitivity and specificity for serotyping (Table 1). PneumoKITy’s output to the serogroup, serotypes that are related serologically use phenotypical typing sera or genogroup, a group of strains that have related capsular sequences were considered not correct in reference to microarray results.

**TABLE 1:** Sensitivity analysis of PneumoKITy’s abilities to detect serotypes using varying levels of specificities^*a*^

	PneumoKITy results (*P* = 90%)				PneumoKITy results (*P* = 80%)				PneumoKITy results (*P* = 70%)				
Sample	no.								no.				
ID	of st	st no. 1 (%)	st no. 2 (%)	st no. 3 (%)	no. of st	st no. 1 (%)	st no. 2 (%)	st no. 3 (%)	of st	st no. 1 (%)	st no. 2 (%)	st no. 3 (%)	st no. 4(%)
S01	1	35B/35D (100)			2	35B/35D (92.65)	**6A_6B_6C_6D (7.35)^*c*^**		2	35B/35D (92.65)	6A_6B_6C_6D (7.35)		
S02	1	35B/35D (100)			1	35B/35D (100)			1	35B (100)			
S03	1	3 (100)			2	3 (78.3)	**13 (21.7)**		2	13 (21.7)	3 (78.3)		
S03.reseq		3 (100)			2	3 (78.82)	13 (21.18)		2	13 (21.18)	3 (78.82)		
S04	1	23A (100)			2	23A (50.54)	**23F (49.46)**		2	23A (50.54)	23F (49.46)		
S05	3	35B/35D (59.3)	14 (27.91)	6A_6B_6C_6D (12.79)	3	35B/35D (59.3)	14 (27.91)	6A_6B_6C_6D (12.79)	4	14 (21.43)	**19B (11.61)**	35B/35D (45.54)	6A_6B_6C _6D(21.43)
S06	2	23F (83.78)	25F_25A_38 (16.22)		2	23F (82.67)	25F_25A_38 (17.33)		2	23F (82.67)	25F_25A_38 (17.33)		
S07	1	23F (100)			1	23F (100)			1	23F (100)			
S08	2	14 (87.72)	23F (12.28)		3	14 (78.12)	23A (10.94)	**23F (10.94)**	3	14 (78.12)	23A (10.94)	23F (10.94)	
S09	1	19F (100)			2	19F (87.23)	**14 (12.77)**		2	14 (12.77)	19F (87.23)		
S09.reseq		14 (13.04)	19F (86.96)		2	19F (86.96)	14 (13.04)		2	14 (13.04)	19F (86.96)		
S10	3	19A_19AF (63.64)	15B/15C (18.18)	1 (18.18)	3	19A_19AF (63.64)	15B/15C (18.18)	1 (18.18)	4	1 (15.38)	15B/15C (15.38)	15F_15A (15.38)	19A_19AF (53.85)
S11	1	15B/15C (100)			2	15B/15C (75.76)	**13 (24.24)**		2	13 (24.24)	15B/15C (75.76)		
S11.reseq		15B/15C (100)			2	15B/15C (75.35)	13 (24.65)		2	13 (24.65)	15B/15C (75.35)		
S12	1	12F (100)			3	12F_12A_12B_44_46 (87.5)	**14 (5.56)**	**6A_6B_6C_6D (6.94)**	4	12F_12A_12B_44_46 (80.77)	14 (5.13)	**28F/28A (7.69)**	6A_6B_6C _6D (6.41)
S13^*b*^	1	22A (100)			1	22A (100)			2	22A (48.54)	22F (51.46)^*c*^		
S14	1	35B/35D (100)			1	35B/35D (100)			1	35B/35D (100)			
S15	2	23F (54.29)	19F (45.71)		2	23F (54.29)	19F (45.71)		3	19F (30.77)	23A (32.69)	23F (36.54)	
S16^*b*^	2	7C (85.51)	19F (14.49)		3	**7B/40 (45.67)**	7C (46.46)	19F (7.87)	3	19F (7.87)	7B/40 (45.67)	7C (46.46)	
S16.reseq^*b*^		7C (87.1)	19F (12.9)		3	7B/40 (46.32)	7C (46.75)	19F (6.93)	3	19F (6.93)	7B/40 (46.32)	7C (46.75)	
S17	1	Serogroup_6_(6E) (100)			1	Serogroup_6_(6E) (100)			1	Serogroup_6_(6E) (100)			
S18	1	14 (100)			2	**13 (57.5)**	14 (42.5)		2	13 (57.5)	14 (42.5)		
S19	1	7C (100)			1	7C (100)			3	**3 (57.14)**	**7B/40 (21.43)**	7C (21.43)	
S19.reseq		7C (100)			1	7C (100)			3	**3 (58.33)**	**7B/40 (20.83)**	7C (20.83)	
S20	1	12F (100)			1	12F (100)			1	12F (100)			
S21	2	16F (50.0)	34 (50.0)		2	16F (50.0)	34 (50.0)		3	16F (27.91)	3 (44.19)	**34(27.91)**	
S22	1	19B (100)			1	19B (100)			1	19B (100)			
S22.reseq		19B (100)			1	19B (100)			1	19B (100)			
S23	2	34 (86.76)	10B (13.24)		2	10B (13.24)	10B (13.24)		3	10A (11.69)	10B (11.69)	34 (76.62)	
S24	1	23B (100)			1	23B (100)			1	23B (100)			

^*a*^ Abbreviations: SP, *S. pneumoniae*; no., number; st, serotype; MM, mixture modeling; cat, category.

^*b*^ Co-carriage with non-*S. pneumoniae* (*B. infantis* and *S. mitis, oralis,* and *parasanguinis*).

^*c*^ Bold text inciate additional serotypes detected from increased sequencing depth. Underlined text indicates additional false-positive serotypes detected with decrease specificity.

### Sensitivity of genomic serotyping

The serotyping results from SeroCall and PneumoKITy were compared to microarray serotyping. The genomic serotyping methods were compared to microarray, and sensitivity was defined as being able to detect the serotype and not just the serogroup level [sensitivity = true positives/(true positives + false negatives)]. Non-typeables were included in the sensitivity calculation. Sensitivity was reported as a percentage with a 95% binomial confidence interval (95% CI).

### Identifying serotypes based on SNP density distributions

Variant calling format files containing the density and frequency of SNPs identified in each sample were generated using Freebayes v1.3.2 (28). They were then visualized using LoFreq v2 (28), which plots the relative density and frequency of observed SNPs relative to the reference genome. The number of local maxima was first estimated by visual inspection of these plots. Each local maximum observed within SNP density plots represents a serotype under the assumption that mutations from the same serotypes occur at the same relative frequencies. A single local maximum in the density plot and a band at 100% relative frequency represent a single serotype from a single sample carriage, and multiple local maxima along the distribution represent multiple serotypes from a single sample. The sum of the local maxima should equal one. The highest local maxima were assumed to be the serotype with the largest relative abundance that was detected by microarray and so on with subsequent local maxima and relative abundances. Sensitivity was reported as a percentage with a 95% CI. This analysis provides an alternative method of assessing multi-carriage that is not dependent on searching for mapped reads or k-mers from a reference database such as SeroCall and PneumoKITy.

We also used a mixture modeling approach instead of visual inspection to estimate the number of serotypes based on the SNP density distributions. This was carried out in R software (version 4.2.2) using the package *gamlss.mx* (version 4.3–5). The modeling approach fitted one-to-size normal distributions to the SNP’s density data for serotype number estimates, and the best estimates were assessed using Akaike Information Criterion values. SNPs were filtered to increase the genomic signal prior to serotyping: SNPs that occurred at 100% frequency were removed, as these were not informative for intra-host diversity. An SNP density threshold was set to exclude SNPs with densities <0.3 due to low-frequency SNPs that could have been a result of potential sequencing artefacts. This was determined by visual inspection of all 24 samples where the areas under the curve were commonly observed between two local maxima.

## RESULTS

### Sample description

Among the 24 samples, microarray detected co-carriage of one to six unique Sp serotypes (Table S1) of which, two samples, S13 and S16, as determined by the microarray, were co-colonized by other non-*pneumoniae* streptococcal species including *Streptococcus infantis* and *Streptococcus mitis*, *Streptococcus oralis*, and *Streptococcus parasanguinis*. The microarray results were used as a point of comparison to assess the sensitivity of the genomic serotyping methods.

### Sequencing results

The average sequencing coverage for the 24 samples was 93% (range 88%–98%), with a corresponding average sequencing depth of 57X (standard deviation, ±17X). Sample S22 had the lowest average depth at 21X (Table S1; Fig. S1). The average percentage of reads that matched the pneumococcal genome was 81% (standard deviation, ±10%). Three samples had more than 20% of reads that did not match that of *S. pneumoniae*, S22, and S13 and S16; the latter two were co-colonized with non-*S. pneumoniae* bacterial species. S22 had 40% of read match with Sp, while the remainder were mostly non-pneumoniae *Streptococcus* (7%), *Lactococcus* (24%), Enterococcaceae (21%), and unclassified (2%). S13 had 86% of reads that matched with *S. pneumoniae*, while the remainder (14%) of the reads were non-pneumoniae *Streptococcus*, and S16 had 62% of the reads matched with *S. pneumoniae*, and the remainder were mostly non-pneumoniae *Streptococcus* and *Actinobacteria* (7%), Eukaryota (1%), and unclassified (3%).

### Sensitivity of genomic serotyping methods compared to microarray

**Fig 1:**
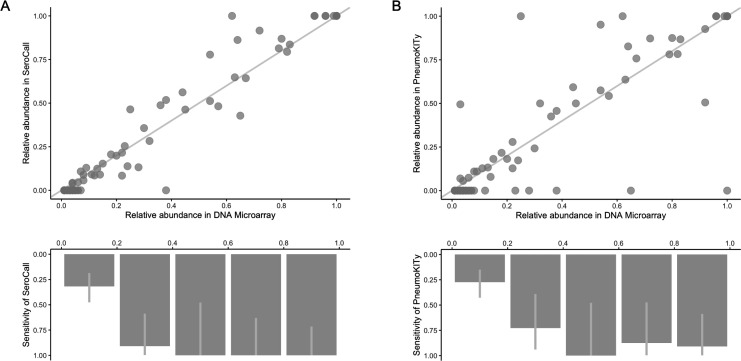
(**A**) Top: relative abundance of 77 serotypes detected by microarray (*x*-axis) and their relative abundances observed by SeroCall (*y*-axis). The distance from the diagonal line represents the extent of discordance; points below the diagonal line are samples with higher relative abundance by microarray, and points above are samples with lower relative abundance by microarray. (**A**) Bottom: SeroCall sensitivity (%) to identify serotypes detected by microarray, regardless of their relative abundance that SeroCall observed, with the light gray line representing a 95% binomial confidence interval. (**B**) Same as (**A**) but using PneumoKITy as the genomic serotyping method.

Microarray detected 77 total pneumococcal serotypes, excluding two [36-like* (S13) and 7F-like* (S16)], which were due to carriage of non-*S. pneumoniae* species containing *cps* genes (Table S1). Of the 77 remaining serotypes, 42 occurred at high abundance (>10%), while the remaining 35 occurred at low abundance (<10%). Of the total 77 serotypes detected by microarray, SeroCall was able to identify 41 [98% sensitivity (95% CI, 0.87–1.00)] at high abundance, 7 [20% (0.08–0.36)] at low abundance, and 48 [62% (0.50–0.72)] at any abundance (Fig. 1).

Compared to microarray, PneumoKITy with an 80% k-mer percentage cut-off, was able to identify the dominant serotype in 22/24 samples (92%) and identified co-carriage with a mix of up to three serotypes. In comparison to the 77 unique serotypes detected by microarray, PneumoKITy was able to identify 36 [86% (95% CI, 0.72–0.95)] at high abundance, six [17% (95% CI, 0.06–0.32)] at low abundance, and 42 [55% (0.42–0.65)] at any abundance (Fig. 1). Of the six serotypes that were unobserved by PneumoKITy at high abundances, two were serotype 3, one was 17F, one was 19B, one was 6B, and one was non-typeable-2.

A subset of samples was resequenced at a higher depth to improve serotype detection sensitivity. Overall, increasing the sequencing depth of the six re-sequenced samples by threefold on average had no impact on the sensitivity of the genomic serotyping methods (Table 2). The specificities of the resequenced samples were reiterated through genomic serotyping using both SeroCall and PneumoKITy where samples maintained the same pneumococcal serotype mixtures and abundance.

**TABLE 2:** Effect of increased sequencing depth on the sensitivity of genomic serotyping methods^*a*^

			SeroCall						PneumoKITy (*P* = 80%)				
Sample	Fold increase	Fold increase	no.	no. cap					no.				
ID	no. of reads	Mean seq depth	of st	reads	st no. 1 (%)	st no. 2 (%)	st no. 3 (%)	st no. 4 (%)	of st.	st no. 1 (%)	st no. 2 (%)	st no. 3 (%)	st no. 4 (%)
S03	Ref	Ref	2	638	03 (79.4)	13 (20.6)			2	3 (78.3)	13 (21.7)		
S03.reseq	3.8	3.7	2	2,561	03 (78.1)	13 (21.9)			2	3 (78.82)	13 (21.18)		
S03.pooled	4.7	4.8	2	3,198	03 (78.0)	13 (22.0)			2	3 (78.86)	13 (21.14)		
S09	Ref	Ref	2	584	19F (91.6)	14 (8.4)			2	19F (87.23)	14 (12.77)		
S09.reseq	2.0	2.0	2	1,098	19F (92.4)	14 (7.6)			2	19F (86.96)	14 (13.04)		
S09.pooled	3.0	3.1	2	1,692	19F (93.3)	14 (6.7)			2	19F (87.67)	14 (12.33)		
S11	Ref	Ref	2	402	15B/15C (64.3)	13 (35.7)			2	15B/15C (75.76)	13 (24.24)		
S11.reseq	4.3	4.2	2	1,706	15B/15C (70.5)	13 (29.5)			2	15B/15C (75.35)	13 (24.65)		
S11.pooled	5.3	5.4	3	2,110	15B/15C (68.8)	13 (28.6)	**21 (2.7)**		2	15B/15C (75.98)	13 (24.02)		
S16^*b*^	Ref	Ref	3	1,148	07C (77.8)	17F (13.2)	19F (9.0)		3	7B/40 (45.67)	7C (46.46)	19F (7.87)	
S16.reseq^*b*^	1.9	1.8	3	2,033	07C (79.2)	17F (13.4)	19F (7.3)		3	7B/40 (46.32)	7C (46.75)	19F (6.93)	
S16.pooled^*b*^	2.9	3.1	3	3,182	07C (80.4)	17F (12.4)	19F (7.2)		4	7B/40 (42.49)	7C (42.96)	19F (5.63)	**17F (8.92)**
S19	Ref	Ref	3	541	07C (46.4)	03 (42.8)	38 (10.8)		1	7C (100)			
S19.reseq	1.1	1.1	3	520	07C (28.2)	03 (64.9)	38 (6.8)		1	7C (100)			
S19.pooled	2.2	2.4	4	1,062	07C (37.2)	03 (48.7)	38 (8.8)	**11A (5.4)**	3	7C (75.56)	**38 (15.56)**	**11A/11D (8.89)**	
S22	Ref	Ref	1	411	19B (100.0)				1	19B (100)			
S22.reseq	3.0	3.0	1	1,183	19B (100.0)				1	19B (100)			
S22.pooled	4.0	4.3	1	1,594	19B (100.0)				1	19B (100)			

^*a*^ Abbreviations: Sp, *S. pneumoniae*; no., number; st, serotype; Ref, the original sequencing run, which the resequenced and pooled samples are compared to regarding sequencing quality. Bold text indicates additional serotypes detected from increased sequencing depth.

^*b*^ Co-carriage with non-*S. pneumoniae* (*B. infantis* and *S. mitis, oralis,* and *parasanguinis)*.

Pooling the original and resequenced samples to further increase the sequencing depth by fourfold on average increased the sensitivity of the genomic serotyping methods to further identify low-abundance serotypes. Across the six samples, microarray detected 20 non-unique serotypes, of which seven were detected at low abundances. SeroCall was able to find an additional two serotypes at low abundances, serotype 21 at 2.7% for sample S11 and serotype 11A at 5.4% for sample S19. PneumoKITy was also able to identify one additional serotype at high abundance, serotype 38 at 15.56%, and two low abundance serotypes, 11A/11D at 8.89% for sample S19 and serotype 17F at 8.92% for sample S16 (Table 2).

### Serotype identification from SNP density

**Fig 2:**
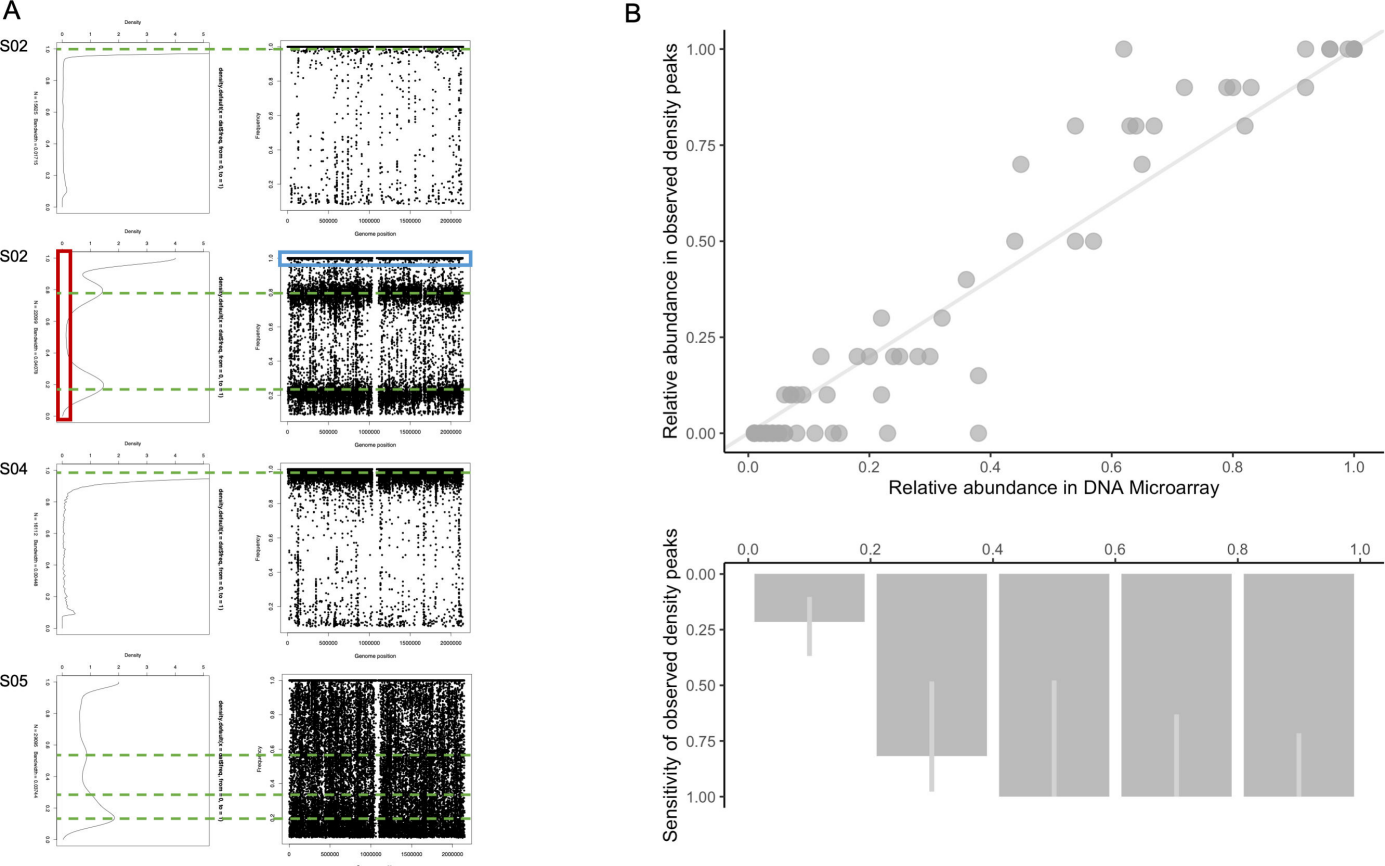
(**A**) Examples of density plots of SNP (left) and frequency plots of SNPs in reference to KK0981 whole genome (right), where a single point is a mutation, and the position along the *y*-axis is the frequency of the mutation relative to the reference genome. The green dotted lines represent local maxima in the SNP density distribution as identified by visual inspection. S02 shows evidence that the individual was infected with a single haplotype, with the widest SNP frequency band positioned near 100%. S03 shows evidence to support that the individual is infected with two haplotypes present at 20% and 80% frequencies. S04 is an example where there is evidence that there is probably a single population; however, there are some signals represented by the small local maxima indicating potential unobserved minor variants. S05 is an example of clear co-carriage; however, it is difficult to distinguish the local maxima. The red box, in the density plot, highlights the threshold (<0.3) that was set to minimize potential artifacts due to sequencing error. The blue box, in the frequency plot, highlights the SNPs that occur at a frequency of 100%, which are SNPs that are present in both the sample and reference genome. (**B**) Top: relative abundance of 77 serotypes detected by microarray (*x*-axis) and likely corresponding local maxima in the SNP density distribution (*y*-axis). Bottom: observed local maxima sensitivity (%) to identify serotypes detected by microarray. The light gray line represents a 95% binomial confidence interval.

For many samples, the density distribution of polymorphic sites showed clear and distinct local maxima of SNP densities (e.g., S03) that likely indicate distinct serotypes and potentially different capsular serotypes (Fig. 2A). However, some samples had less discernible local maxima in the SNP density distribution (e.g., S05) or no clearly defined local maxima but some indication of the presence of minority serotypes indicated by a wider band of SNPs just below 100% than typical for single serotype samples (e.g.. S04 vs S02).

For all 24 samples, the range of visually observable local maxima ranged from one to three with two being observed the most often (Fig. S2). Compared to the number of serotypes detected by microarray, 5/24 samples had the same number of local maxima, all four single serotype carriage samples, and a sample with two types carried at high abundance. The number of local maxima identified visually in the density plots for the remainder of 19 of the 24 samples was, on average, two fewer serotypes detected than by microarray (Table 3). Local maxima detection in the SNP density had 88% sensitivity (95% CI, 0.74–0.96) for identifying likely corresponding serotypes at high abundance, 14% (0.05–0.30) at low abundance, and 55% (0.43–0.66] at any abundance (Fig. 2B).

**TABLE 3:** Sensitivity of SeroCall and PneumoKITy compared to microarray^*a*^

	SNP frequency			SeroCall						PneumoKITy (*P* = 80%)			
Sample	Visual no.	% peaks	MM no.	no.						no.			
ID	of peaks	occur at	of st	of st	st no. 1 (%)	st no. 2 (%)	st no. 3 (%)	st no. 4 (%)	st no. 5 (%)	of st	st no. 1 (%)	st no. 2 (%)	st no. 3 (%)
S01	2	90%, 10%	2	1	35B (100)					2	35B/35D (92.65)	6A_6B_6C_6D (7.35)	
S02	1	100%	1	1	35B (100)					1	35B/35D (100)		
S03	2	80%, 20%	3	2	03 (79.4)	13 (20.6)				2	3 (78.3)	13 (21.7)	
S04	1	100%	2	1	23A (100)					2	23A (50.54)	23F (49.46)	
S05	3	50%, 30%, 20%	3	5	35B(56.2)	14 (21.6)	06E[6A] (9.1)	19B (8.5)	21 (4.6)	3	35B/35D (59.3)	14 (27.91)	6A_6B_6C_6D (12.79)
S06	2	80%, 20%	3	2	23F (86.2)	38 (13.8)				2	23F (82.67)	25F_25A_38 (17.33)	
S07	1	100%	3	1	23F (100)					1	23F (100)		
S08	2	90%, 10%	5	3	14 (81.4)	23F (12.9)	23A (5.7)			3	14 (78.12)	23A (10.94)	23F (10.94)
S09	2	90%, 10%	3	2	19F (91.6)	14 (8.4)				2	19F (87.23)	14 (12.77)	
S10	2	80%, 20%	3	3	19A (64.8)	15B/15C (19.9)	01 (15.3)			3	19A_19AF (63.64)	15B/15C (18.18)	1 (18.18)
S11	2	80%, 20%	6	2	15B/15C (64.3)	13 (35.7)				2	15B/15C (75.76)	13 (24.24)	
S12	2	90%, 10%	2	3	12F (86.9)	28F (8.9)	14 (4.3)			3	12F_12A_12B_44_46 (87.5)	14 (5.56)	6A_6B_6C_6D (6.94)
S13^*b*^	1	100%	1	1	22A (100)					1	22A (100)		
S14	1	100%	1	1	35B (100)					1	35B/35D (100)		
S15	2	50%, 15%	4	2	19F (51.8)	23F (48.2)				2	23F (54.29)	19F (45.71)	
S16^*b*^	2	80%, 20%	3	3	07C (77.8)	17F (13.2)	19F (9.0)			3	7B/40 (45.67)	7C (46.46)	19F (7.87)
S17	1	100%	1	1	06E[6B] (100)					1	Serogroup_6_(6E) (100)		
S18	3	50%, 40%, 10%	3	2	13 (51.2)	14 (48.8)				2	13 (57.5)	14 (42.5)	
S19	3	70%, 20%, 10%	3	3	07C (46.4)	03 (42.8)	38 (10.8)			1	7C (100)		
S20	1	100%	1	1	12F (100)					1	12F (100)		
S21	2	70%, 30%	4	3	16F (46.3)	34 (28.3)	03 (25.4)			2	16F (50.0)	34 (50.0)	
S22	1	100%	2	1	19B (100.0)					1	19B (100)		
S23	2	90%, 10%	2	3	34 (83.6)	10B (12.4)	19F (4.0)			2	34 (86.76)	10B (13.24)	
S24	1	100%	1	1	23B (100.0)					1	23B (100)		

^*a*^ Abbreviations: *Streptococcus pneumoniae* (Sp), number (no.), serotype (st), mixture modelling (MM), category (cat).

^*b*^ Co-carriage with non-*Streptococcus pneumoniae (Bifidobacterium infantis,* and *Streptococcus mitis, oralis,* and *parasanguinis).*

Of the original 24 samples, mixture modeling identified between one to six serotypes from the SNP frequencies and estimated the same number of serotypes as microarray in 6 (25%), more in 5 (21%), and fewer in 13 (54%) (Table 3).

The resequenced samples with an increased sequencing depth had a qualitative impact on the SNP frequencies for three of the six samples. Samples S03, S11, and S22 demonstrate darker frequency bands in the resequenced runs, which highlight repeated SNP detection; however, the same number of frequency bands remain, indicating that the sensitivity has not been impacted (Fig. S3). The remaining three samples maintained quantitatively similar frequency distributions. The mixture modeling on the resequenced samples revealed that S03 and S19 maintained the same number of serotypes, and S09, S11, and S22 reduced the number of serotypes by one, while S16 increased the serotype from three to seven (Table 3).

### Sensitivity analyses

For PneumoKITy, configuring the alternative filter cut-off value for the k-mer percentage parameter from the default (90%) to 80% resulted in higher sensitivity for identifying serotypes without compromising specificity. The adjustment increased sensitivity to identify an additional 10 serotypes across nine samples. However, lowering the threshold to 70% lowered the specificity, and thus, false-positive serotypes were observed (Table 1).

Co-carriage detection sensitivity was cross-validated using the pipeline implemented by Tonkin-Hill et al. (29), which combines SeroCall with a deconvolution strategy using the SeroBA algorithm (30). While the results largely aligned (Table S2), the combined approach of Tonkin-Hill et al., detected six additional strains that were undetected by SeroCall. However, these were all designated “untypeable,” suggesting that the underlying strains were non-typeable or that there was insufficient read coverage of the serotype locus to provide a classification.

## DISCUSSION

Detecting co-carriage of Sp serotypes using whole-genome sequencing can be advantageous in providing additional information, e.g., on phylogenetic relationships and antimicrobial resistance. Detection of low-abundance carriage can be important to our understanding of the ecological niche that allows the co-existence of pneumococci and reservoirs for serotype replacement. In reference to the microarray results, both genomic pneumococcal serotyping methods, SeroCall and PneumoKITy, reliably identified serotypes present at high abundance (>10%) among samples with multiple serotype carriage, with a reduced sensitivity among serotypes carried at low relative abundance (<10%). However, we demonstrate that increasing sequencing depth can increase the sensitivity of these methods in identifying low-abundance serotypes. Additionally, the SNP density distributions largely corresponded to the relative serotype abundance identified by microarray with potential future use in haplotype reconstruction using the associated reads that contain the SNPs at the relevant frequencies.

SeroCall identified the dominant serotypes in all 24 samples. However, PneumoKITy did not identify the major populations in two samples, one of which was identified at the serogroup level and the other an undetected serotype 3. The developers of PneumoKITy, Sheppard et al., noted that there is a limitation in identifying serotype 3, particularly in co-carriage at low abundances and that this could be potentially mitigated by lowering the specificity parameter. In our study, serotype 3 was co-carried at a high abundance and was only observable when the k-mer percentage threshold was lowered from 80% to 70%. The 80% threshold resulted in 100% specificity across the study samples. However, false-positive serotypes were observed when the threshold was lowered from 80% to 70%.

Most of the discordance between microarray and genomic methods was due to the genomic serotyping methods lacking sensitivity to identify serotypes at relatively low abundance, highlighting the potential importance of read depth in the genomic detection of multiple serotype carriage. Increasing sequencing depth resulted in increased sensitivity; however, there remained instances where PnuemoKITy was also not able to identify non-dominant high-abundance serotypes. This observation may be explained by the reference database used by the program lacking a sufficient number of reference sequences that are reflective of current circulating diverse strains in Africa (23). Despite this, SeroCall and PneumoKITy are free-access options with sufficient sensitivity for routine carriage surveillance to characterize dominant serotypes; additionally, SeroCall can identify co-carriage of serotypes > 10% relative abundance.

An advantage of this study was the inclusion of the two pneumococcal samples containing non-*S. pneumoniae* bacterial species reflecting the natural complexity of nasopharyngeal samples, which can contain other species that can grow on the streptococcal-selective culture medium. The inclusion of these samples revealed observable non-*S. pneumoniae* sequences detected, 36-like for S13 and 7F-like for S16, which impacted the sensitivity of the genomic serotyping methods, particularly for PneumoKITy where the dominant serotype 17F was unobserved. Non-pneumococcal species carrying related capsule gene sequences can be a source of false-positive serotyping results as demonstrated by methods such as PCR (31). However, the non-pneumococcal serotypes, 36-like and 7F-like, were detected from a panel of streptococcal species-specific genes plus the detection of related but incomplete *cps* loci, which have been previously isolated and sequenced, confirming the presence of related *cps* genes and *cps* loci in other streptococcal species (32). This provides confidence that microarray is able to detect and distinguish non-pneumococcal “serotypes” effectively.

Previous studies have evaluated serotyping methods but have been limited to single-colony picks or have compared genomic methods without reference to microarray. Sheppard et al. did include a small comparison between PneumoKITy and SeroCall; however, it was limited to a small number of unique serotypes (*n* = 10), while our study had 34 unique serotypes (23). Similarly, a study by Swarthout et al., from which our microarray data set was a subset, observed a high concordance in serotype identification between latex agglutination (using single-colony picks), genomic serotyping (PnuemoCaT), and microarray using 1,347 samples from community carriage surveillance in Blantyre, Malawi (10). Manna et al. observed discordant results between PneumoCaT, seroBA, and SeroCall, as well as discordant results within SeroCall in the identification of single carriage of serotype 14-like, lacking serotype 14 capsule, identifying them as serotype 14 and/or non-typeable (33), highlighting the importance of additional phenotypic testing to validate serotyping data. Tonkin-Hill et al. implemented a different approach using deep sequencing to capture the within-host genetic diversity and observed a doubled increase in sensitivity of detecting serotype 1 compared to the gold standard approach (29). Additionally, they identified more serotypes among co-carriage compared to latex sweep methods highlighting the sensitivity compared to alternative serotyping approaches.

Knight et al. highlighted that read depth would affect the sensitivity of SeroCall and recommended that samples should have between 2 and 3 million reads per sample (24). In our study, only five of the 30 sequenced samples had >1 million Sp reads. We re-sequenced and pooled six samples to increase the depth to an average of 196X (from 45X). This led to the identification of previously undetected serotypes with SeroCall and PneumoKITy, most of which were present at low abundances. These results concur with the notion that higher sequencing depth could improve sensitivity for identifying co-carriage of low abundant serotypes from genomic data, but SeroCall still had high sensitivity for detecting co-carriage in a setting with limited capacity. Additional studies are required to determine the necessary sequencing depth to detect serotypes detected at 1% relative abundance.

The genomic serotyping tools available to detect multiple serotypes lack sensitivity for detecting serotypes carried in low relative abundance when compared to microarray, potentially due to the limited sequencing depth. Despite that, there is an added benefit to including sequencing as a part of routine surveillance, including additional information for phylogenetic inference to investigate transmission dynamics. Tonkin-Hill et al. demonstrated the high sensitivity of genomic serotyping of pneumococcal co-carriage and highlighted the added insights on drug resistance and within-host evolution (29). Capturing within-host diversity can improve inference on transmission links (34), and only considering the dominant variant can substantially underestimate the number of transmission links (29). More specifically, the inability to detect serotypes at low abundances would result in underestimating the within-host genetic diversity and thus impact the ancestral state reconstruction resulting in more ambiguous subtrees reconstructed. Additionally, reconstructing the haplotypes detected in co-carriage would help us better understand the role of minor variants in pneumococcus transmission dynamics.

### Study limitations

The first limitation of this study is the small sample size of 24, which limited the variation in combination and the quantity of co-carriage we were able to study. The second limitation is the use of a single next-generation sequencing method (Illumina MiSeq). Other sequencing methods could result in a higher depth of coverage or longer sequencing reads, which could impact the sensitivity and specificity of the genomic serotyping. Increasing sequencing depth would increase the sensitivity of identifying low-abundance variants as we observed when we pooled the duplicate sequencing runs together, which improved our identification of serotypes <10%. Additionally, we suspect that increasing the read lengths would improve the alignment, thus increasing specificity using the genomic serotyping methods. The third limitation is the potential degradation of the DNA between the sample preparation for microarray and whole-genome sequencing resulting in potentially less optimal sequencing data. The fourth limitation of genomic serotyping methods is the potential bias induced by the reference database used in the pipeline, which could lead to misclassification or incorrect quantification. For example, closely related serotypes would be difficult to quantify compared to distantly related serotypes. Finally, estimating the number of serotypes by visual local maxima from the density plots can be subjective and can lead to underestimations due to potentially overlapping maxima or serotypes at relatively similar abundances. Additionally, the highest local maxima are assumed to correspond with the serotype at the largest relative abundance detected by microarray, and this cannot currently be validated; thus, this assumption results in unobserved serotypes at relatively low abundances.

### Conclusion

SeroCall can detect co-carriage at increased sensitivity and specificity compared to PneumoKITy, while PuemoKITy is less computationally intensive with high sensitivity with an 80% k-mer percentage cut-off, which can be more beneficial in large-scale surveillance studies. Regardless, both methods offer an increasingly effective method to detect the occurrence of co-carriage with the added benefit of allowing further analyses into population structure, antimicrobial resistance, and phylogenetic inference to investigate transmission dynamics.

## Data Availability

The whole-genome sequencing data has been made available for download on the European Nucleotide Archive under study accession “ PRJEB79259” including the corresponding sample alias that are referenced in this manuscript. Bioproject: Phylogenetic inference of pneumococcal transmission from cross-sectional data, a pilot study. Accession number: PRJEB79259.

## SUPPLEMENTAL MATERIAL

Figure S1 spectrum.03643-23-s0001.tiff Sequencing depth for all of the 24 original sequenced samples.

Supplemental figure captions spectrum.03643-23-s0002.docx Fig. S1-S3 captions.

Figure S2 spectrum.03643-23-s0003.tiff Density (left) and frequency (right) plots for the original 24 sequences.

Figure S3 spectrum.03643-23-s0004.tiff Frequency plots of SNPs where a single point represents a single polymorphic site to the reference genome.

TABLE S1 spectrum.03643-23-s0005.docx Overview of quality of WGS reads and the microarray results on serotypes detected.

TABLE S2 spectrum.03643-23-s0006.docx Serotypes detected from methods used by Tonkin-Hill et al.

ASM does not own the copyrights to Supplemental Material that may be linked to, or accessed through, an article. The authors have granted ASM a non-exclusive, world-wide license to publish the Supplemental Material files. Please contact the corresponding author directly for reuse.
