# Supplementary figures and images for: Evaluating methods for identifying and quantifying Streptococcus pneumoniae co-colonization using next-generation sequencing data
Source: Microbiol Spectr. 2024 Nov 5;12(12):e03643-23. doi: 10.1128/spectrum.03643-23 (PMC11619295; doi:10.1128/spectrum.03643-23)

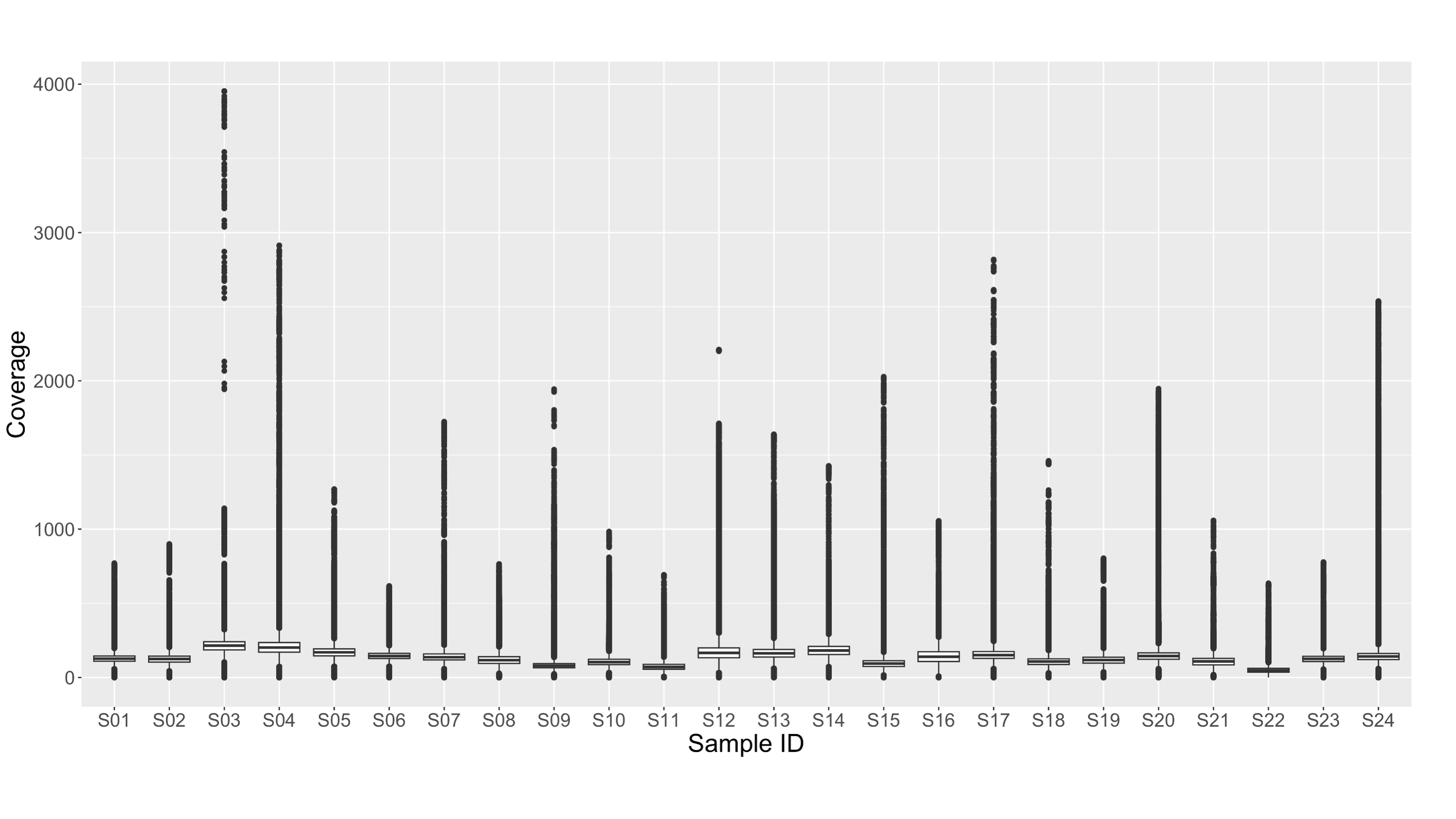

Supplement: Figure S1 — Sequencing depth for all of the 24 original sequenced samples. [file spectrum.03643-23-s0001.tiff]

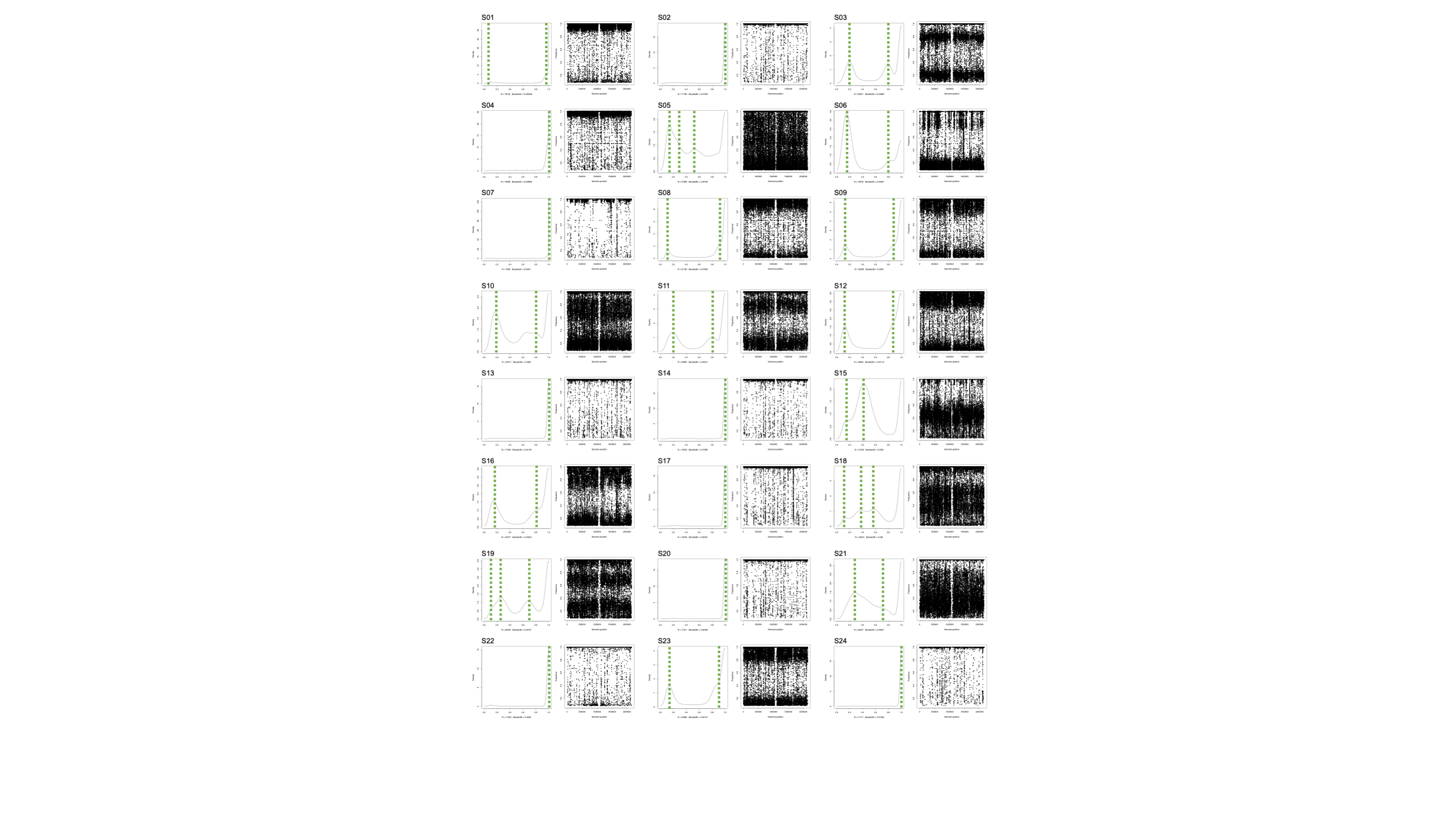

Supplement: Figure S2 — Density (left) and frequency (right) plots for the original 24 sequences. [file spectrum.03643-23-s0003.tiff]

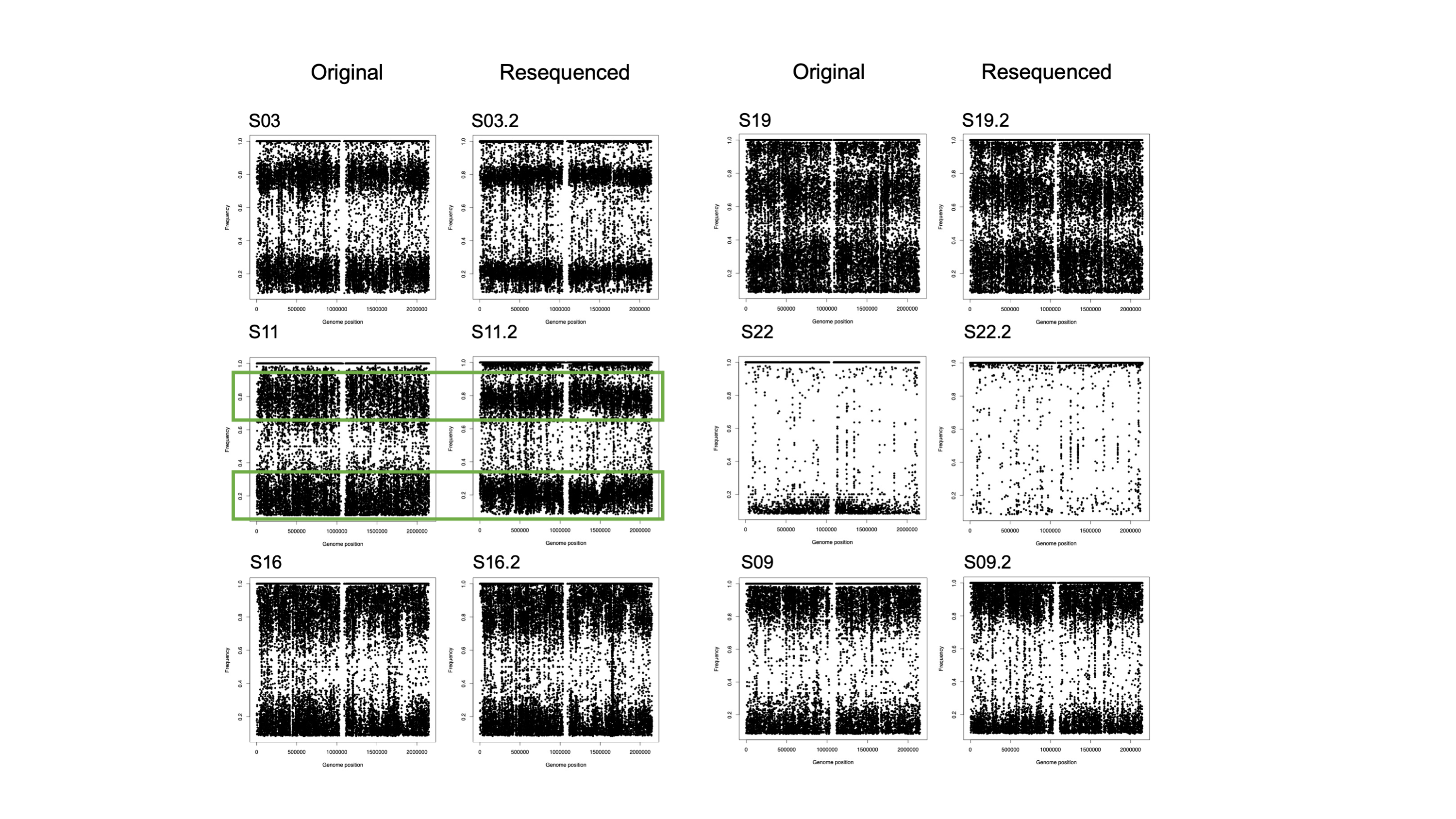

Supplement: Figure S3 — Frequency plots of SNPs where a single point represents a single polymorphic site to the reference genome. [file spectrum.03643-23-s0004.tiff]
