# Supplementary material for: Evaluating methods for identifying and quantifying Streptococcus pneumoniae co-colonization using next-generation sequencing data
Source: Microbiol Spectr. 2024 Nov 5;12(12):e03643-23. doi: 10.1128/spectrum.03643-23 (PMC11619295; doi:10.1128/spectrum.03643-23)
Supplement: Supplemental figure captions — Fig. S1-S3 captions. [file spectrum.03643-23-s0002.docx]

Supplemental Figure 1. Sequencing depth for all of the 24 original sequenced samples. Boxes and whiskers display the sequencing depth's lower, median, and upper bounds.

Supplemental Figure 2. Density (left) and frequency (right) plots for the original 24 sequences based on SNPs identified relative to the reference genome. The green dotted lines represent the frequencies observed in the density plots.

Supplemental Figure 3. Frequency plots of SNPs where a single point represents a single polymorphic site to the reference genome. Original and resequenced plots are displayed side by side for all six samples, the average increase in mean sequencing depth was 3-fold. The green box highlights the darker SNP frequency bands between the original vs the resequenced run for S11.
