# Supplementary material for: Evaluating methods for identifying and quantifying Streptococcus pneumoniae co-colonization using next-generation sequencing data
Source: Microbiol Spectr. 2024 Nov 5;12(12):e03643-23. doi: 10.1128/spectrum.03643-23 (PMC11619295; doi:10.1128/spectrum.03643-23)
Supplement: TABLE S1 — Overview of quality of WGS reads and the microarray results on serotypes detected. [file spectrum.03643-23-s0005.docx]

|  |  |  |  |  | DNA Microarray Results | | | | | | |
| --- | --- | --- | --- | --- | --- | --- | --- | --- | --- | --- | --- |
| Sample | Number of | Sequencing | Mean | % Matched | No. |  |  |  |  |  |  |
| ID | mapped reads | coverage | depth | Sp reads | of st | st no. 1 (%) | st no. 2 (%) | st no. 3 (%) | st no. 4 (%) | st no. 5 (%) | st no. 6 (%) |
| S01 | 675683 | 92 | 51 | 81 | 3 | **35B (92)** | 6A/B[6A] (6) | 14 (2) |  |  |  |
| S02 | 639079 | 91 | 53 | 87 | 1 | **35B (100)** |  |  |  |  |  |
| S03 | 1053737 | 92 | 86 | 86 | 2 | **3 (82)** | **13 (18)** |  |  |  |  |
| S04 | 1031636 | 94 | 88 | 86 | 4 | **23A (92)** | 20A/B[20B] (4) | 23F (3) | 15F (1) |  |  |
| S05 | 892799 | 96 | 74 | 83 | 6 | **35B (44)** | **14 (22)** | **19B (12)** | **6A/B[6A] (11)** | 21 (6) | NT2 (5) |
| S06 | 785997 | 98 | 67 | 84 | 5 | **23F (64)** | **38 (24)** | NT3b (6) | NT2 (5) | 9L-like (1) |  |
| S07 | 776809 | 97 | 65 | 82 | 2 | **23F (96)** | 11A/D/E[11A] (4) |  |  |  |  |
| S08 | 586890 | 93 | 48 | 83 | 4 | **14 (79)** | 23F (9) | 23A (8) | 19A (4) |  |  |
| S09 | 450136 | 93 | 34 | 80 | 6 | **19F (72)** | **14 (22)** | 22F (3) | 35B (1) | 19B (1) | NT2 (1) |
| S10 | 561528 | 95 | 45 | 82 | 5 | **19A (63)** | **15B/C[15B] (20)** | **1 (15)** | 11A/D/E[11A] (1) | 23B-like (1) |  |
| S11 | 409984 | 93 | 32 | 81 | 3 | **15B/C[15B] (67)** | **13 (30)** | 21 (3) |  |  |  |
| S12 | 914487 | 95 | 71 | 82 | 5 | **12F/44[12F] (80)** | 28A/F[28F] (8) | 20A/B[20B] (5) | 14 (4) | 6A/B[6B] (3) |  |
| S13* | 819610 | 90 | 65 | 79 | 2* | **22A (96)** | 18B/C[18C] (1) | 36-like* (3) |  |  |  |
| S14 | 977902 | 90 | 79 | 86 | 2 | **35B (99)** | 38 (1) |  |  |  |  |
| S15 | 506404 | 98 | 41 | 82 | 4 | **23F (57)** | **19F (38)** | 11A/D/E[11A] (3) | 6A/B[6B] (2) |  |  |
| S16* | 701352 | 92 | 56 | 62 | 3* | **40/7B/7C[7C] (54)** | **17F (28)** | **19F (14)** | 7F-like* (4) |  |  |
| S17 | 792903 | 93 | 66 | 81 | 1 | **6A/B[6B] (100)** |  |  |  |  |  |
| S18 | 556650 | 94 | 44 | 84 | 5 | **13 (54)** | **14 (36)** | 3 (7) | 34 (2) | 38 (1) |  |
| S19 | 623422 | 95 | 51 | 85 | 4 | **3 (65)** | **40/7B/7C[7C] (25)** | 38 (7) | 11A/D/E[11A] (3) |  |  |
| S20 | 748438 | 90 | 62 | 86 | 1 | **12F/44[12F] (100)** |  |  |  |  |  |
| S21 | 573636 | 95 | 47 | 85 | 3 | **16F (45)** | **34 (32)** | 3 (23) |  |  |  |
| S22 | 273984 | 90 | 21 | 40 | 2 | **19B (62)** | **NT2 (38)** |  |  |  |  |
| S23 | 655286 | 94 | 55 | 84 | 3 | **34 (83)** | **10B (13)** | 19F (4) |  |  |  |
| S24 | 745529 | 88 | 60 | 83 | 1 | **23B-like (100)** |  |  |  |  |  |

Table S1. Overview of quality of WGS reads and the microarray results on serotypes detected.

Abbreviations: Streptococcus pneumoniae (Sp), number (no.), serotype (st)

Bold text indicates serotypes detected by DNA microarray at high (>10%) relative abundance

Underlined text indicates samples co-colonised by non-Streptococcus pneumoniae species

*Co-carriage with non-Streptococcus pneumoniae (Bifidobacterium infantis, and Streptococcus mitis, oralis, and parasanguinis)
