# Supplementary material for: Evaluating methods for identifying and quantifying Streptococcus pneumoniae co-colonization using next-generation sequencing data
Source: Microbiol Spectr. 2024 Nov 5;12(12):e03643-23. doi: 10.1128/spectrum.03643-23 (PMC11619295; doi:10.1128/spectrum.03643-23)
Supplement: TABLE S2 — Serotypes detected from methods used by Tonkin-Hill et al. [file spectrum.03643-23-s0006.docx]

| Sample ID | mGEMS/seroba | SeroCall |
| --- | --- | --- |
| S01 | untypable, 35B | 35B |
| S02 | 35B, untypable | 35B |
| S03 | untypable, 03 | 03, 13 |
| S04 | 23A, untypable | 23A |
| S05 | 6E(6A), untypable, 35B, 19B, 14 | 35B, 14, 06E(6B), 19B, 21 |
| S06 | 38, 23F, untypable | 23F, 38 |
| S07 | 23F | 23F |
| S08 | untypable, 14 | 14, 23F |
| S09 | untypable, 19F | 19F, 14 |
| S10 | untypable, 19A, 01 | 19A, 15B/15C, 01 |
| S11 | 15C, untypable | 15B/15C, 13 |
| S12 | untypable, 12F | 12F, 28F |
| S13* | 22A | 22A |
| S14 | 35B | 35B |
| S15 | untypable, 23F, 19F | 19F, 23F |
| S16* | 19F, 17F, 07C | 07C, 17F, 19F |
| S17 | 6E(6B), untypable | 06E(6B) |
| S18 | 14, 13 | 14, 13 |
| S19 | 03, untypable, 07C | 03, 07C, 38 |
| S20 | 12F | 12F |
| S21 | 16F, 03, 34 | 16F, 34, 03 |
| S22 | untypable | 19B |
| S23 | untypable, 34, 10B | 34, 10B, 19F |
| S24 | 23B | 23B |

Table S2. Serotypes detected from methods used by Tonkin-Hill et al. for cross- validation
